# Measurement-Error Analysis of Fiber Bragg Grating Flexible Sensor for Displacement-Field Monitoring of Geotechnical Engineering

**DOI:** 10.3390/s22197168

**Published:** 2022-09-21

**Authors:** Changbin Tian, Xiangxue Ma, Bo Peng, Xin Ma, Zhiyu Li

**Affiliations:** 1 School of Information and Electrical Engineering, Shandong Jianzhu University, Jinan 250101, China; 2 Shandong Tianrui Heavy Industry Co., Ltd., Weifang 261061, China

**Keywords:** strain-transfer rate, displacement reconstruction algorithm, arrangement interval of sensing points, measurement error

## Abstract

Monitoring geotechnical structures and providing real-time early warning is a key measure to mitigate the impacts of disasters (slope slip, subsidence, dam deformation, bridge settlement, etc.). The fiber Bragg grating (FBG) flexible sensor, developed by the combination of flexible material and an FBG sensor, is widely used in geotechnical engineering health monitoring due to its excellent performance. The flexible sensor can perform regional and quasi-distributed measurements of the displacement field of the measured structure, and accurately reflect the operating state of the engineering structure. However, in practical engineering applications, factors such as the strain-transfer rate between the flexible substrate and sensing points, the displacement reconstruction algorithm, and the arrangement interval of the sensing points can cause measurement error, which, in turn, leads to a decrease in the displacement-measurement accuracy. In this paper, the following analysis is performed by means of theoretical derivation and model establishment. The influence of the length, width, and thickness of the cemented layer, the shear modulus of the flexible substrate, and the radius of the groove on the strain-transfer rate were analyzed, and the referential parameters were determined. The displacement reconstruction algorithm is essentially a recursive algorithm, which inevitably introduces cumulative error; the relationship between the layout interval of the sensing points and the measurement error is discussed. Considering the fabrication cost of the sensor and the allowable range of error, a sensing-point-layout interval of 100 mm was chosen. The feasibility and effectiveness of the simulation theory were verified by carrying out deformation-sensing experiments on the developed FBG flexible sensor. The research results can theoretically guide the packaging and fabrication of the FBG flexible sensor, thereby improving the measurement accuracy of the flexible sensor for the measured structure.

## 1. Introduction

Geotechnical engineering has the characteristics of a complex operating environment, wide distribution area, and long service time. Affected by factors such as rainfall, seismic loads, and overloading, a series of engineering disasters, such as slope slippage, the subsidence of the subgrade, the deformation of dams, and the settlement of bridges, have occurred, which have caused a lot of loss of life and property [1,2,3]. Therefore, monitoring engineering structures to provide real-time early warning is a key measure to reduce the impacts of disasters.

Geotechnical engineering is conducted under complex geological conditions and in harsh operating environments, and the initial stages of engineering structural diseases are concealed, and so it is challenging to monitor it in real time [4,5]. A series of monitoring instruments have been elaborated over the past decades for the measurement of engineering displacement fields. In terms of two-dimensional deformation-field measurement, such as slope slippage, subgrade subsidence, and bridge deformation, the main measurement instruments include inclinometers [6], LVTDs (linear variable differential transformers) [7], inductive displacement sensors [8], and multipoint extensometers [9]; GPS technology [10] and laser scanning technology [11] are mainly used to measure the three-dimensional deformation fields of dam reservoirs. However, the traditional instruments have single-measurement parameters and low precision, and they are active devices that are susceptible to electromagnetic interference and poor waterproof and moisture-proof performances. Therefore, they are not competent for the tasks of the long-term measurement of the engineering structure deformation field and the provision of real-time early warning. Compared with traditional measuring instruments, fiber Bragg grating (FBG) sensors have the advantages of anti-electromagnetic interference, high measurement accuracy, a long service life, and easy installation, and they are waterproof and moisture-proof [12,13,14]. Therefore, as an efficient health monitoring instrument, FBG sensors are widely used in structural-deformation-field measurement. For example, various types of sensors, such as FBG displacement sensors, FBG strain gauges, FBG pressure sensors, FBG tension sensors, FBG vibration sensors, and FBG inclination sensors, have been designed to measure the mechanical parameters in real time, such as the displacement, strain, pressure, force, vibration, and inclination of engineering structures, and to then conduct a safety assessment of the structure. However, most FBG sensors only use the point-based monitoring of the parameters related to the stability of engineering structures, which are not suitable for the measurement of the overall displacement field of the structure and the location of the deformation surface.

The FBG flexible sensor has been widely used in engineering monitoring, and it is fabricated by combining FBG sensors with flexible materials, with good waterproof, moisture-proof, and recovery properties after bending, by means of surface bonding or grooved bonding [15,16]. The flexible substrate package is applied to protect the fragile FBG sensing points. In the production process of the FBG flexible sensor, it should be noted that the force must be uniform throughout the sensing points to prevent the reflection spectrum from being distorted [17]. It is important to select a suitable grating length, which affects the reflectivity of the reflection spectrum [18]. The cross influence of strain and temperature on the central wavelength of the sensing point is considered. It is necessary to take certain measures to eliminate the influence of temperature on the central wavelength to improve the measurement accuracy of the displacement field [19]. FBG flexible sensors overcome the deficiency of point sensors in monitoring the deformation fields of engineering structures, and they can perform regional and quasi-distributed measurements of the two-dimensional and three-dimensional deformation fields of structures [20,21], which more accurately reflect the safety status of engineering structures. In the process of geotechnical engineering monitoring, the FBG flexible sensor is embedded in the interior of the measured structure, and the strain data measured by the flexible sensor are converted into displacement information through the displacement reconstruction algorithm to obtain the displacement deformation of the measured structure, and finally realize the real-time monitoring of the health state of the engineering structure.

However, in practical engineering applications, factors such as the strain-transfer rate between the flexible substrate and sensing points, the displacement reconstruction algorithm, and the arrangement interval of the sensing points will cause measurement errors. The span of geotechnical engineering structures is large, and the errors will gradually accumulate, which leads to a reduction in the displacement-measurement accuracy. However, how the above factors affect the reconstruction error of the deformation field has not been reported. In this paper, a strain-transfer model between the flexible substrate and sensing points is established. The effects of the length, width, and thickness of the cemented layer, the shear modulus of the flexible substrate, and the radius of the groove on the strain-transfer rate were analyzed by simulation, and referential parameters were given. The displacement reconstruction algorithm is essentially a recursive algorithm, which inevitably introduces accumulated error. The flexible sensor model was established, and the relationship between the layout interval of the sensing points and the measurement error was analyzed. Considering the fabrication cost of the sensor and the allowable range of error, a sensing-point-layout interval of 100 mm was chosen. The research results theoretically guide the packaging and fabrication of the FBG flexible sensor, thereby improving the measurement accuracy of the flexible sensor for the measured structure.

## 2. Displacement Sensing Principle of Geotechnical Engineering Structure

**Figure 1:**
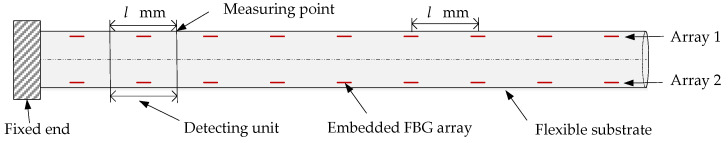
Schematic diagram of FBG flexible sensor.

As shown in Figure 1, the commonly used flexible sensor bonds the sensing points on the surface or groove of the flexible substrate at an interval (*l*). The purpose of multiarray sensing-point layout is to eliminate the influence of temperature on the central wavelength and improve the detection accuracy. The sensing point and measuring point are located at the center and end of the detecting unit with the length *l*, respectively. The flexible sensor developed by the combination of flexible material and an FBG sensor has the advantages of flexible and diverse structural forms. The sensors are arranged according to the measurement requirements, and they can conveniently monitor the measured structure. The measurement principle is as follows: When the measured structure is deformed, the flexible sensor deforms cooperatively because the stiffness of the flexible substrate is far less than that of the environmental media. When the flexible substrate is deformed, its surface-strain value is transmitted to the sensing point through the shear stress of the cemented layer. According to the FBG sensing detection principle and the pure bending theory of flexible beams in material mechanics [22], the strain value at the detecting unit is calculated from the wavelength change of the sensing point. A displacement reconstruction algorithm is used to obtain the displacement of each measuring point. Then, the overall deformation shape of the flexible sensor can be achieved, which can reflect the displacement deformation of the measured structure [23]. However, the strain-transfer rate between the flexible substrate and sensing points, the displacement reconstruction algorithm, and the layout interval of the sensing points affect the reconstruction accuracy of the bending shape of the flexible sensor. The influence of each factor on the accuracy of the displacement reconstruction will be introduced in detail.

## 3. Error Analysis of Displacement-Field Reconstruction

### 3.1. Analysis of Strain-Transfer Mechanism

The combination of flexible materials and FBG sensors generally adopts two methods: surface bonding and grooved bonding. In the following, the effects of different factors on the strain-transfer rate between the flexible substrate and sensing point under the two bonding methods will be analyzed.

#### 3.1.1. Analysis of Surface-Bonded Strain-Transfer Mechanism

**Figure 2:**
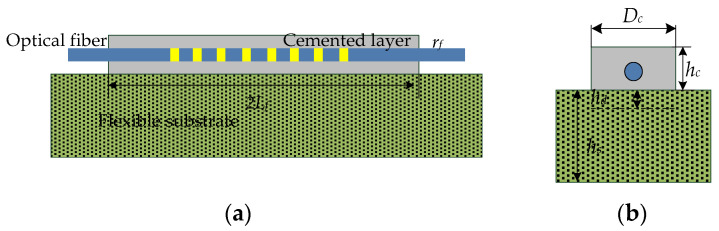
Schematic diagram of surface-bonded strain-transfer model: (**a**) front view; (**b**) side view.

The surface-bonded strain-transfer model is shown in Figure 2. The optical fiber is bonded on the surface of the flexible substrate through a colloid. The transfer model is a three-layer structure of flexible substrate, a cemented layer, and optical fiber. When the flexible substrate is deformed, its surface strain causes the cemented layer to strain through the shear stress between the substrate and cemented layer. At the same time, the shear stress between the cemented layer and fiber interface causes the fiber to strain. The strain-transfer rate between the substrate and optical fiber is affected by the length (2*L_f_*), width (*D_c_*), thickness (*h_c_*), and other factors of the cemented layer. It is assumed that the thickness of the flexible substrate is *h_s_*, and the influence depth of the cemented layer on the substrate is *h_d_*. To facilitate the derivation of the strain-transfer model between the substrate, cemented layer, and optical fiber, it is assumed that the physical properties and material properties of the core and cladding of the optical fiber are the same, and there is no slippage between the flexible substrate, the cemented layer, and the optical fiber. The strain between each layer is within the elastic range, and the strain gradient is the same; the influences of external factors, such as temperature, humidity, pH, etc., are not considered.

**Figure 3:**
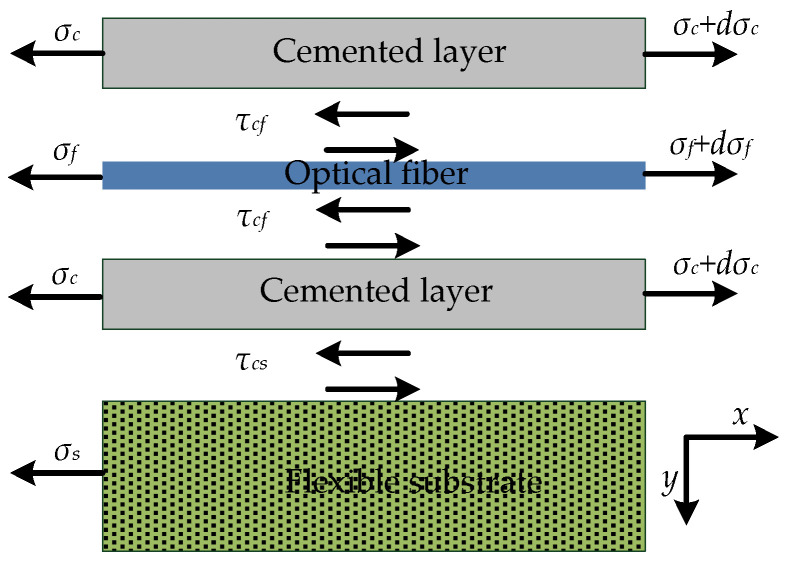
Schematic diagram of strain transfer between different layers in surface-bonded model.

The strain-transfer model between layers is shown in Figure 3, where *E*, *G*, *τ*, *σ*, *ε*, and *u* are specified to represent the elastic modulus, shear modulus, shear stress, axial stress, strain, and displacement, respectively. The subscripts *f*, *c*, and *s* represent the optical fiber, cemented layer, and flexible substrate, respectively. The shear stresses between the cemented layer and optical fiber, and between the cemented layer and flexible substrate, are *τ_cf_* and *τ_cs_*, respectively. A coordinate system was established, and the element (*dx*) of each layer was taken for the force analysis.

Because there is no slip between the three layers of the flexible substrate, cemented layer, and optical fiber, the strains between each layer are within the elastic range and the strain gradient is the same. Therefore, the stress-balance equation of the optical fiber is:

(1) $$d\sigma_{f}(x)\pi r_{f}^{2}-2\pi r_{f}\tau_{cf}(x)dx=0$$

and the stress-balance equation of the cemented layer is:

(2) $$d\sigma_{c}(x)(D_{c}h_{c}-\pi r_{f}^{2})+2\pi r_{f}\tau_{cf}(x)dx+\tau_{cs}(x)D_{c}dx=0$$

Simultaneous Equations (1) and (2) can obtain:

(3) $$\tau_{cs}(x)=-\frac{d\sigma_{c}(x)}{dx}(h_{c}-\frac{\pi r_{f}^{2}}{D_{c}})-\frac{d\sigma_{f}(x)}{dx}\frac{\pi r_{f}^{2}}{D_{c}}$$

Because the strain gradients of the optical fiber and cemented layer are the same, the following can be obtained:

(4) $$\left\{ \begin{array}{l} \frac{d\sigma_{f}(x)}{dx}=E_{f}\frac{d\varepsilon_{f}(x)}{dx}\\ \frac{d\sigma_{c}(x)}{dx}=E_{c}\frac{d\varepsilon_{c}(x)}{dx} \end{array}\right.$$

(5) $$\tau_{cf}(x)=\frac{E_{f}r_{f}}{2}\frac{d\varepsilon_{f}(x)}{dx}$$

(6) $$\tau_{sc}(x)=-[E_{c}(h_{c}-\frac{\pi r_{f}^{2}}{D_{c}})+E_{f}\frac{\pi r_{f}^{2}}{D_{c}}]\frac{d\varepsilon_{f}(x)}{dx}$$

The shear stress between the layers varies linearly. When *y* = *h_c_* + *h_d_*, *τ_s_*(*x*) = 0, and when *y* = *h_c_*, *τ_s_*(*x*) = *τ_sc_*(*x*), the shear stress of the flexible substrate can be achieved:

(7) $$\tau_{s}(x)=-(h_{c}+h_{d}-y)\frac{1}{h_{d}}[E_{c}(h_{c}-\frac{\pi r_{f}^{2}}{D_{c}})+E_{f}\frac{\pi r_{f}^{2}}{D_{c}}]\frac{d\varepsilon_{f}(x)}{dx}$$

The shear stress of the flexible substrate can also be expressed as:

(8) $$\tau_{s}(x)=G_{s}\frac{du(x)}{dy}$$

After simultaneous Equations (7) and (8), calculate the integral of *y* and then the derivative of *x* to obtain the strain of the flexible substrate:

(9) $$\varepsilon_{s}(x)=\varepsilon_{c}(x)-\frac{1}{2}\frac{h_{d}}{G_{s}}[E_{c}(h_{c}-\frac{\pi r_{f}^{2}}{D_{c}})+E_{f}\frac{\pi r_{f}^{2}}{D_{c}}]\frac{d^{2}\varepsilon_{f}(x)}{dx^{2}}$$

Calculate the cemented-layer strain in the same way:

(10) $$\varepsilon_{c}(x)=\varepsilon_{f}(x)-\frac{E_{f}}{G_{c}}(\frac{\pi r_{f}^{2}}{D_{c}}+\frac{r_{f}}{2})(\frac{h_{c}}{4}+\frac{r_{f}}{2})\frac{d^{2}\varepsilon_{f}(x)}{dx^{2}}$$

The regulations are as follows:

(11) $$\frac{1}{k_{s}^{2}}=\frac{E_{f}}{G_{c}}(\frac{\pi r_{f}^{2}}{D_{c}}+\frac{r_{f}}{2})(\frac{h_{c}}{4}+\frac{r_{f}}{2})+\frac{1}{2}{\frac{h_{d}}{G_{s}}}_{}[E_{c}(h_{c}-\frac{\pi r_{f}^{2}}{D_{c}})+E_{f}\frac{\pi r_{f}^{2}}{D_{c}}]$$

Equation (10) is simplified as:

(12) $$\frac{d^{2}\varepsilon_{f}(x)}{dx^{2}}-k_{s}^{2}\varepsilon_{f}(x)=-k_{s}^{2}\varepsilon_{s}(x)$$

Formula (12) is the strain differential equation between the flexible substrate and optical fiber, *k_s_* is the sensing hysteresis factor, and there is no strain transmission at the end of the optical fiber and bonding layer. Therefore, the ratio of the axial strain of the optical fiber to the strain of the flexible substrate is:

(13) $$\frac{\varepsilon_{f}(x)}{\varepsilon_{s}(x)}=1-\frac{\cosh(k_{s}x)}{\cosh(k_{s}L_{f})}$$

Therefore, the average strain-transfer coefficient between the flexible substrate and optical fiber is:

(14) $$\alpha_{s}=\frac{{\bar{\varepsilon }}_{f}(x)}{\varepsilon_{s}(x)}=\frac{2\int_{0}^{L_{f}}\varepsilon_{f}(x)dx}{2L_{f}\varepsilon_{s}(x)}=1-\frac{\sinh(k_{s}L_{f})}{k_{s}L_{f}\cosh(k_{s}L_{f})}$$

It can be seen from the above formula that the length (2*L_f_*) of the cemented layer and the hysteresis factor (*k_s_*) (determined by the parameters of the optical fiber, cemented layer, and flexible substrate) are the main factors that affect the average strain-transfer coefficient. To analyze the influence characteristics of various physical parameters on the average strain-transfer coefficient (*α_s_*), the single-mode silica fiber (elastic modulus: *E_f_* = 72 GPa; radius: *r_f_* = 0.0625 mm) was selected in this paper, and the value ranges of the physical parameters are shown in Table 1.

(1) Influence of cemented-layer length (2*L_f_* ) on average strain-transfer coefficient (*α_s_*)

**Figure 4:**
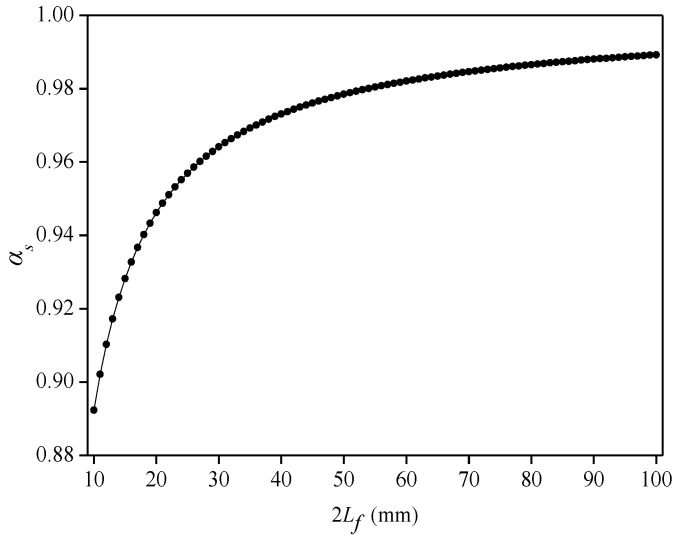
Relationship between length of cemented layer and average strain-transfer coefficient in surface-bonded model.

The sensing point is bonded on the surface of the flexible substrate. First, the influence of the length (2*L_f_*) of the cemented layer on the average strain-transfer coefficient (*α_s_*) is analyzed. When calculating, set *D_c_* = 3 mm, *h_c_* = 0.8 mm, *E_c_* = 1.5 GPa, *G_c_* = 0.7 GPa, and *G_s_* = 0.8 GPa. As shown in Figure 4, as the length of the cemented layer increases, the value of the *α_s_* also increases gradually. When 2*L_f_* = 10 mm, *α_s_* = 0.89, and when the bond length is greater than 22 mm, the average strain-transfer coefficient is greater than 0.95. During the fabrication of the flexible sensor, the length of the cemented layer should be increased as much as possible when the installation space allows, thereby improving the strain-transfer rate between the sensing point and flexible substrate.

(2) Influence of cemented-layer width (*D_c_*) on average strain-transfer coefficient (*α_s_*)

**Figure 5:**
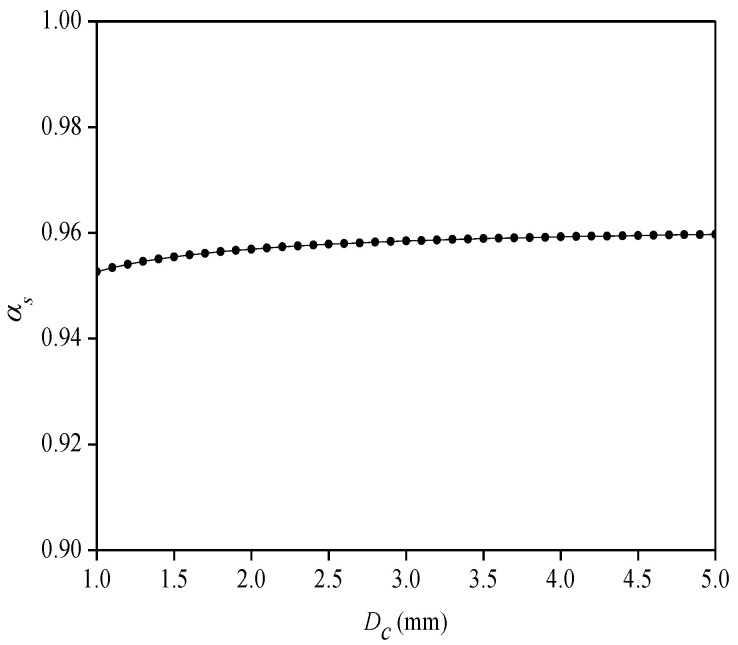
Relationship between width of cemented layer and average strain-transfer coefficient.

For the effect of the width (*D_c_*) of the cemented layer on the *α_s_*, set 2*L_f_* = 50 mm, *h_c_* = 0.8 mm, *E_c_* = 1.5 GPa, *G_c_* = 0.7 GPa, and *G_s_* = 0.8 GPa. As shown in Figure 5, the *α_s_* value gradually increases with the increase in the width of the cemented layer. When *D_c_* = 1 mm, *α_s_* = 0.95, and when *D_c_* = 5 mm, *α_s_* = 0.96, and so the width of the cemented layer has little effect on the average strain-transfer coefficient. The width of the cemented layer can be appropriately increased to ensure a firm adhesion between the flexible substrate and sensing point. However, if the cemented layer is too wide, then it will affect the bending characteristics of the flexible substrate, and so the width of the cemented layer is generally about 3 mm.

(3) Influence of cemented-layer thickness (*h_c_*) on average strain-transfer coefficient (*α_s_*)

**Figure 6:**
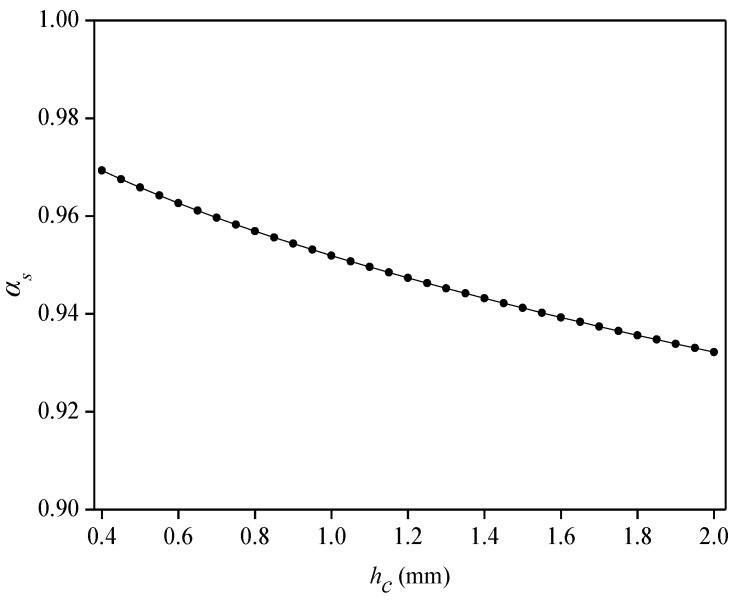
Relationship between thickness of cemented layer and average strain-transfer coefficient.

For the influence of the thickness of the cemented layer on the *α_s_*, let 2*L_f_* = 50 mm, *D_c_* = 3 mm, *E_c_* = 1.5 GPa, *G_c_* = 0.7 GPa, and *G_s_* = 0.8 GPa. As shown in Figure 6, the value of the *α_s_* gradually decreases with the increase in the thickness of the cemented layer. When *h_c_* = 0.4 mm, *α_s_* = 0.97, and when *h_c_* = 2 mm, *α_s_* = 0.93, and so the thickness of the cemented layer can be appropriately reduced during the sensor fabrication process to improve the strain-transfer rate. Due to the harsh working environment of the flexible sensor, if the thickness of the adhesive is too thin, then the sensing point will easily fall off the flexible substrate. The thickness of the adhesive layer is about 1 mm when the sensor is fabricated.

(4) Influence of shear modulus (*G_s_*) of flexible substrate on average strain-transfer coefficient (*α_s_*)

**Figure 7:**
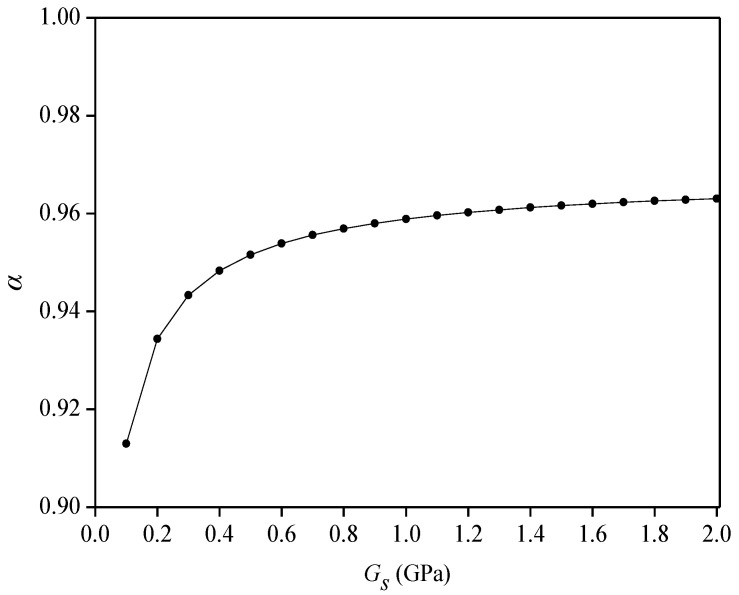
Relationship between shear modulus and average strain-transfer coefficient.

A material with high strength, corrosion resistance, and good flexibility was selected as the flexible substrate. To study the effect of the shear modulus of the substrate on the *α_s_*, set 2*L_f_* = 50 mm, *D_c_* = 3 mm, *h_c_* = 0.8 mm, *E_c_* = 1.5 GPa, and *G_c_* = 0.7 GPa. As shown in Figure 7, as the shear modulus of the flexible substrate increases, the value of the *α_s_* also increases gradually. When *G_s_* = 0.1 GPa, *α_s_* = 0.91; when *G_s_* = 2 GPa, *α_s_* = 0.96; when *G_s_* is greater than 0.5 GPa, the *α_s_* value increases slowly. The shear modulus of the flexible substrate selected in the actual sensor fabrication is about 1 GPa.

#### 3.1.2. Analysis of Grooved-Bonded Strain-Transfer Mechanism

**Figure 8:**
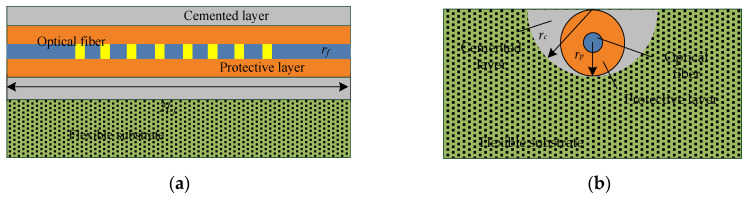
Schematic diagram of grooved-bonded strain-transfer model: (**a**) front view; (**b**) side view.

When the FBG flexible sensor monitors the deformation field of geotechnical engineering, its working environment is complex and harsh, and the sensing point is fragile and easily damaged. To meet the long-term-monitoring task of the sensor in a rough environment, the optical fiber is coated with polymer materials (such as acrylate, polyimide, etc.) to improve the strength and toughness of the optical fiber. The surface of the flexible substrate is grooved along the axis direction, and the optical fiber containing the coating layer (protective layer) is bonded in the groove of the flexible substrate. The sensing point is encapsulated and protected by the grooving. The grooved-bonded strain-transfer model is shown in Figure 8. Different from the surface-bonded model, this model is a four-layer structure of an optical fiber, a protective layer, a cemented layer, and a flexible substrate.

**Figure 9:**
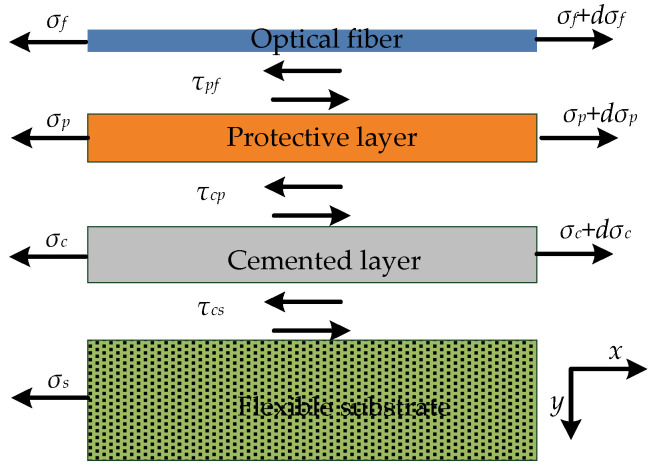
Schematic diagram of strain transfer between different layers in grooved-bonded model.

The strain-transfer model between the layers is shown in Figure 9. It is stipulated that *E*, *G*, *τ*, *σ*, *ε*, and *u* represent the elastic modulus, shear modulus, shear stress, axial stress, strain, and displacement, respectively; the subscripts *p*, *f*, *c*, and *s* represent the protective layer, optical fiber, cemented layer, and flexible substrate, respectively. The coordinate system was established to analyze the force of the four-layer structure of the optical fiber, protective layer, cemented layer, and flexible substrate.

For the force analysis of the optical fiber:

(15) $$\pi r_{f}^{2}[\sigma_{f}(x)+d\sigma_{f}(x)]+2\pi r_{f}dx\cdot \tau_{pf}(x)-\pi r_{f}^{2}\sigma_{f}(x)=0$$

For the force analysis of the protective layer:

(16) $$(\pi r_{p}^{2}-\pi r_{f}^{2})[\sigma_{p}(x)+d\sigma_{p}(x)]+2\pi r_{p}\tau_{pc}dx-\sigma_{p}(x)(\pi r_{p}^{2}-\pi r_{f}^{2})-2\pi r_{f}\tau_{pf}(x)dx=0$$

For the force analysis of the cemented layer:

(17) $$[\sigma_{c}(x)+d\sigma_{c}(x)](\frac{\pi r_{c}^{2}}{2}-\pi r_{p}^{2})+\pi r_{c}\tau_{cs}(x)dx-\sigma_{c}(x)(\frac{\pi r_{c}^{2}}{2}-\pi r_{p}^{2})-2\pi r_{p}\tau_{cp}(x)dx=0$$

Because there is no slip between the layers and the strain gradient is the same, the elastic modulus of the optical fiber is much larger than that of the protective layer. In the same way, the sensing hysteresis factor (*k_g_*) is calculated as follows:

(18) $$\frac{2}{k_{g}^{2}}=E_{f}r_{f}^{2}[\frac{1}{G_{p}}\ln(\frac{r_{p}}{r_{f}})+\frac{1}{G_{c}}\ln(\frac{r_{c}}{\sqrt{2}r_{p}})]$$

Therefore, the average strain-transfer coefficient between the flexible substrate and optical fiber is:

(19) $$\alpha_{g}=1-\frac{\sinh(k_{g}L_{f})}{k_{g}L_{f}\cosh(k_{g}L_{f})}$$

From Equations (14) and (19), it can be seen that the expressions of the average strain-transfer coefficients of the grooved-bonded and the surface-bonded methods are the same, and only the expressions of the sensing hysteresis factor are different. Similarly, the length (2*L_f_*) of the cemented layer and the hysteresis factor (*k_g_*) (determined by the physical parameters of the shear modulus (*G_p_*) of the protective layer, the radius (*r_p_*) of the protective layer, the shear modulus (*G_c_*) of the cemented layer, and the radius (*r_c_*) of the cemented layer) are the main factors that affect the average strain-transfer coefficient. The influence characteristics of the parameters of each physical quantity on the *α_g_* will be analyzed below.

(1) Influence of cemented-layer length (2*L_f_*) on average strain-transfer coefficient (*α_g_*)

**Figure 10:**
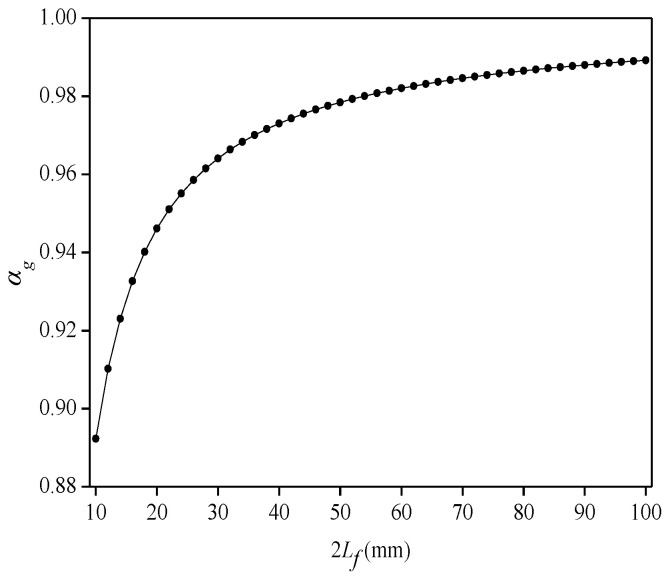
Relationship between length of cemented layer and average strain-transfer coefficient in grooved-bonded model.

The FBG sensing point is bonded in the groove of the flexible substrate, and the influence of the length of the cemented layer on the *α_g_* is studied in the grooved-bonded method, where *G_c_* = 0.7 GPa, *G_p_* = 1 GPa, and *r_c_* = 0.6 mm. As shown in Figure 10, the *α_g_* value gradually increases with the increase in the length of the cemented layer. When 2*L_f_* = 10 mm, *α_g_* = 0.89, and when 2*L_f_* = 100 mm, *α_g_* = 0.99. As with the surface-bonded method, the bonding length of the optical fiber should be increased as much as possible to improve the strain-transfer rate between the optical fiber and flexible substrate.

(2) Influence of groove radius (*r_c_*) on average strain-transfer coefficient (*α_g_*)

**Figure 11:**
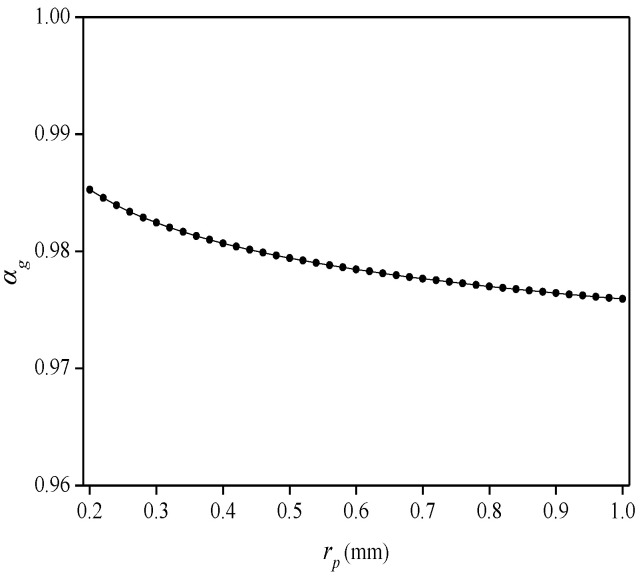
Relationship between radius of groove and average strain-transfer coefficient.

Study the relationship between the groove radius (*r_c_*) and the *α_g_*, and set 2*L_f_* = 50 mm, *G_c_* = 0.7 GPa, and *G_p_* = 1 GPa when calculating. As shown in Figure 11, the value of the *α_g_* decreases very slowly as the groove radius increases. When *r_c_* = 0.2 mm, *α_g_* = 0.985, and when *r_c_* = 1 mm, *α_g_* = 0.976, and so the groove radius has little effect on the *α_g_*. The use of polymer materials (such as acrylate, polyimide, etc.) for coating improves the strength and toughness of the optical fiber while hardly affecting the strain-transfer characteristics [24,25,26]. To facilitate the production of the flexible sensor, a groove with a radius of about 1 mm can be opened on the surface of the substrate along the axis, and the sensing point is bonded in the groove. To make the flexible sensor waterproof and moisture-proof, and to prolong its life, use waterproof glue or the same glue as the substrate material to seal the groove until the surface of the substrate is smooth. The encapsulation method does not change the bending characteristics of the flexible substrate, and it improves the strain-transfer rate between the sensing point and substrate.

### 3.2. Analysis of Displacement Reconstruction Algorithm

**Figure 12:**
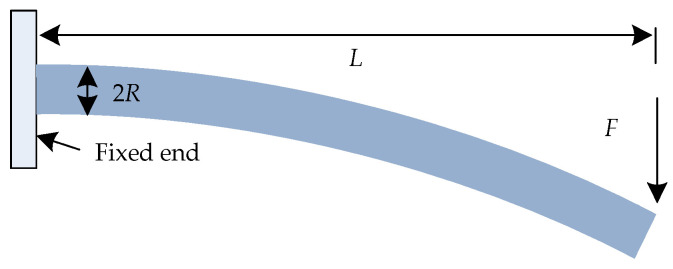
Schematic diagram of cantilever structure under force (*F*).

When the FBG flexible sensor monitors the geotechnical structure, one end is fixed, and its whole can be regarded as a cantilever structure, as shown in Figure 12. It is assumed that the length of the flexible substrate is *L,* and the radius is *R*. From the mechanics of materials [27,28] (when *L*/*R* is much greater than 10, the radial shear stress can be ignored), the surface strain of the substrate under the action of the force (*F*) can be expressed as:

(20) $$\varepsilon =\frac{F(L-x)R}{EI}=\omega^{\prime \prime }R$$

where *E* is the elastic modulus of the material, *I* is the moment of inertia, *x* is the distance between the strain point and fixed end, *ω* is the deflection, and the strain information of the flexible substrate can be realized from the sensing point. When the displacement deformation occurs in the whole or local range of the geotechnical structure, because the stiffness of the flexible substrate is far less than that of the environmental media, the displacement field of the measured structure can be reconstructed based on the strain information of the sensing point and the displacement reconstruction algorithm. Many experts and scholars also have some differences in the structural design of flexible sensors according to different measured structures. However, the essence is to use the strain-displacement reconstruction algorithm to calculate the displacement of each measuring point of the sensor. Assuming that the layout interval of the sensing points is *l*, the displacement of the *n*-*th* measuring point is *S_n_*, and the strain of the detection unit is *ε_n_*, there are mainly the following algorithms for the displacement reconstruction of flexible sensors [29]:

(1) The Simpson integral method:
  (21) $$S_{n}=\frac{l^{2}}{9R}\left[14\sum_{i=0}^{n-1}\varepsilon_{i+l}-24\sum_{i=0}^{n-1}(n-i)\varepsilon_{i+l}-2\sum_{i=1}^{n-1}(6n-6i-1)\varepsilon_{i}-\varepsilon_{n}-(6n+1)\varepsilon_{0}\right]$$
(2) The trapezoidal integral method:
  (22) $$S_{n}=\frac{l^{2}}{8R}\left[\varepsilon_{n}+(2n-3)\varepsilon_{1}+4\sum_{i=2}^{n-1}(n-i)\varepsilon_{i}\right]$$
(3) The difference equation method:
  (23) $$\left[\begin{array}{l} S_{2}\\ \;\vdots \\ S_{n+1} \end{array}\right]=\frac{l^{2}}{R}{\left[\begin{array}{ccccc} 1 & 0 & \cdots & \cdots & 0\\ -2 & \cdots & \cdots & \cdots & \vdots \\ 1 & \ddots & \ddots & \ddots & \vdots \\ \vdots & \ddots & \ddots & \ddots & 0\\ 0 & \cdots & 1 & -2 & 1 \end{array}\right]}_{n\times n}^{-1}\left[\begin{array}{l} \varepsilon_{1}\\ \;\vdots \\ \varepsilon_{n} \end{array}\right]$$
(4) The indefinite beam method:
  (24) $$\left\{ \begin{array}{l} S_{n}=S_{n-1}+v_{n}+\Delta l_{n}\sin\theta_{n}+(l_{n}+\Delta l_{n})\sin(\sum_{i=1}^{n-1}\theta_{i})\\ v_{n}=\frac{l_{n}^{2}}{6R}\frac{3l_{n\varepsilon_{1}}-l_{n}}{l_{n\varepsilon_{2}}-l_{n\varepsilon_{1}}}(\varepsilon_{n2}-\varepsilon_{n1})-\frac{l_{n}^{2}}{2R}\varepsilon_{n1}\\ \tan\theta_{n}=\frac{l_{n}}{R}\frac{l_{n\varepsilon_{2}}-l_{n}/2}{l_{n\varepsilon_{1}}-l_{n\varepsilon_{2}}}(\varepsilon_{n2}-\varepsilon_{n1})-\frac{l_{n}}{R}\varepsilon_{n2} \end{array}\right.$$
(5) The beam element decomposition method:
  (25) $$\left\{ \begin{array}{l} S_{n}=S_{n-1}+L\cdot \sin\sum_{i=1}^{n-1}\theta_{i}+\omega_{n}\cdot \cos\sum_{i=1}^{n-1}\theta_{i}\\ \omega_{n}=\frac{2l^{2}}{3R}\varepsilon_{n}\\ \theta_{n}=\frac{l}{R}\varepsilon_{n} \end{array}\right.$$
(6) The arc-fitting method:
  (26) $$\left\{ \begin{array}{l} S_{n}=O_{\left(n-1\right)}-(\rho_{\left(n-1\right)}-\rho_{n})\cdot \cos\sum_{i=1}^{n-1}\theta_{i}-\rho_{n}\cdot \cos\sum_{i=1}^{n}\theta_{i}\\ \theta_{n}=\frac{l\cdot \varepsilon_{n}}{R}\\ \rho_{n}=\frac{R}{\varepsilon_{n}} \end{array}\right.$$
(7) The conjugate beam method:
  (27) $$S_{n+1}=l^{2}\cdot \left[\sum_{i=1}^{n}\frac{\varepsilon_{i}+\varepsilon_{i+1}}{2R}(\frac{1}{2}+n-i)\right]$$

The above strain-displacement reconstruction algorithms are essentially recursive algorithms. The displacement of the remaining measuring points is gradually deduced from the displacement of the initial point by the known boundary conditions. There are approximate conditions in the recursion process, each step of the operation introduces an error, and the error will gradually accumulate with the derivation process. Therefore, the displacement of the flexible sensor, reconstructed based on the strain information of the sensing points and the displacement reconstruction algorithm, inevitably introduces a certain error.

### 3.3. Simulation Analysis of Layout Interval of Sensing Points

The FBG sensing points are arranged on the flexible substrate at a certain interval, and the arrangement interval also affects the reconstruction accuracy of the displacement field. Taking the two-dimensional displacement field as an example, the finite element simulation method is employed to analyze the influence of the distribution-point interval on the displacement reconstruction accuracy. The flexible-substrate model, with a length of 2000 mm, diameter of 5 mm, and POM (polyformaldehyde) material, was established, in which the elastic modulus and shear modulus are 2.6 GPa and 938 MPa, respectively. In the simulation experiment, the complete mesh of the model is divided into 16,053, the minimum element quality is 0.3781, the elastic model is selected, and the geometric nonlinearity is considered. A constraint was imposed on one end of the flexible-substrate model, and displacements of 150 mm, 50 mm, and 120 mm were applied along the *y*-axis direction at (600, −2.5, 0), (1300, −2.5, 0), and (2000, −2.5, 0), respectively.

**Figure 13:**
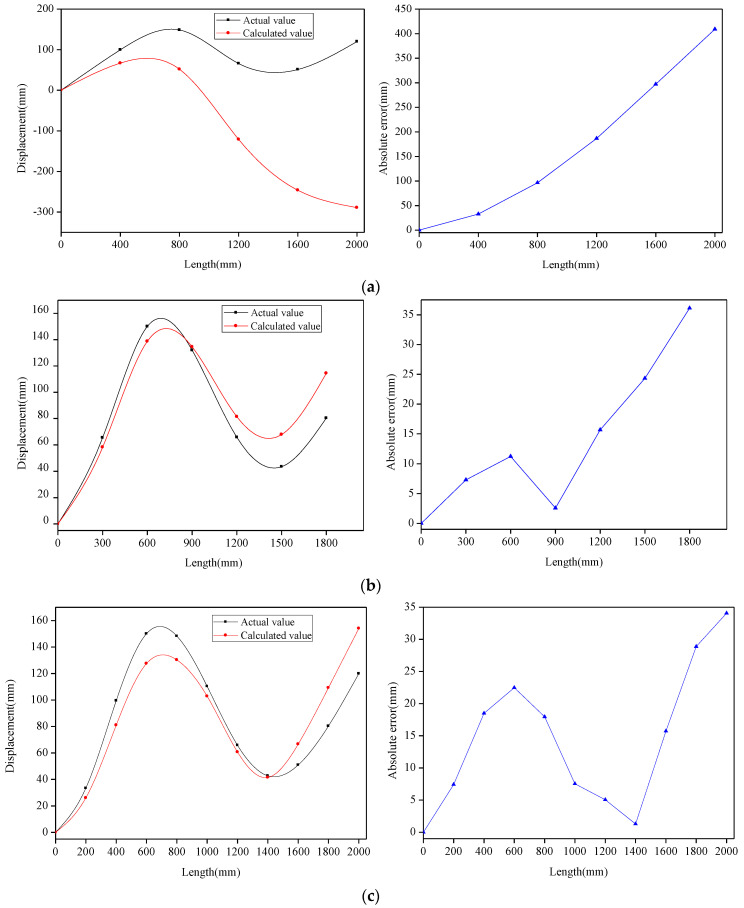

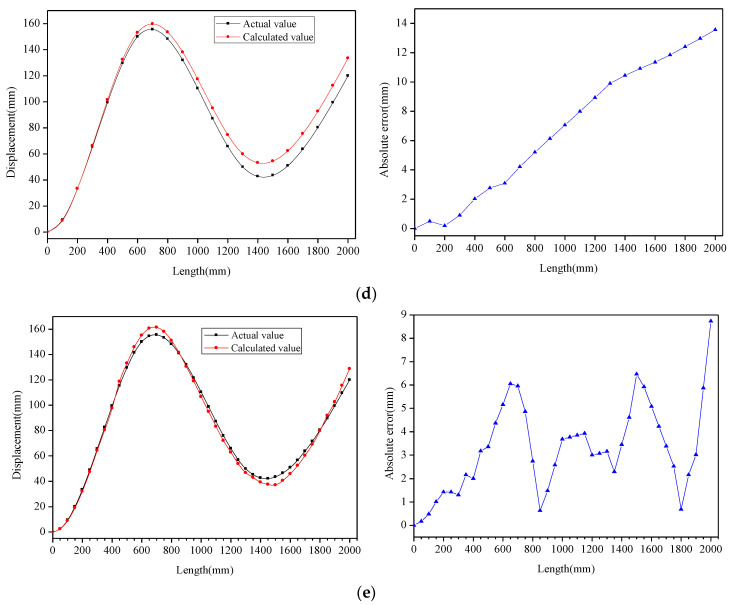
Comparison between calculated displacement and actual displacement of measuring points at different intervals, as well as absolute error of reconstructed measuring points: (**a**) *l* = 400 mm; (**b**) *l* = 300 mm; (**c**) *l* = 200 mm; (**d**) *l* = 100 mm; (**e**) *l* = 50 mm.

Along the *x*-axis direction of the flexible-substrate model, the strain value of the measuring point was extracted with a distance interval of *l*, and the displacement value of the measuring point was extracted as a comparison. Let *l* be 400 mm, 300 mm, 200 mm, 100 mm, and 50 mm, respectively, to simulate the layout of the FBG sensing points during the production of the flexible sensor. The displacement reconstruction algorithm was applied to reconstruct the shape of the flexible-substrate model to achieve the calculated displacement of each measuring point. Figure 13 presents the comparison between the calculated displacement and actual displacement of the measuring points at different intervals, as well as the absolute error of the reconstructed measuring points. It can be seen from the figure that, due to the cumulative error, the measuring points with the largest absolute error appeared at the end. The positive and negative cumulative errors were offset, resulting in the local reduction in the absolute error of the measuring points.

The maximum-absolute-error value and maximum-error percentage of the reconstructed measuring points are shown in Table 2. As can be seen from Table 2, as the interval (*l*) decreases, both the maximum-absolute-error value and maximum-error percentage of the measuring points decrease. Therefore, to improve the measurement accuracy of flexible sensors, the layout interval of the sensing points should be as small as possible. Considering the production cost of the sensor, as well as the strain-transfer rate between the sensing point and flexible substrate (the bonding length of the sensing point), the layout interval of the sensing point in the actual production is generally 100 mm. It can be seen from the figure that when *l* = 100 mm, the absolute error of the measuring point increases gradually, and the average absolute error is 7.12 mm, which can meet the needs of geotechnical engineering structure measurements.

## 4. Experiments and Analysis

### 4.1. Fabrication of FBG Flexible Sensor

**Figure 14:**
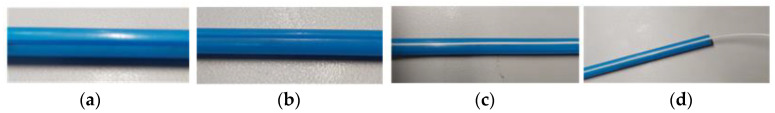
Fabrication process of FBG flexible sensor: (**a**) grooved; (**b**) cleaned; (**c**) protected; (**d**) leading.

During the fabrication of the FBG flexible sensor, a POM rod (consistent with the material in the simulation experiment) with high mechanical strength, fatigue resistance, and a good recovery performance after bending was selected as the flexible substrate. In addition, the POM rod is easy to process, and its working temperature range is from −40 °C to 120 °C. These excellent characteristics make it fully qualified as a substrate for flexible sensors to monitor engineering deformation fields. The diameter of the flexible rod is 5 mm, and the effective sensing length is 2000 mm. At the same time, the FBG array with polyimide coating was selected as the sensing element. Compared with polymer optical fibers, glass optical fibers have a higher resolution, and so they can achieve a higher detection accuracy [30]. The fiber Bragg grating sensors used in this paper were fabricated by the phase-mask method, and the specific parameters are shown in Table 3. The factors that affect the strain-transfer rate between the sensing point and flexible substrate were analyzed in the simulation experiments. To protect the fragile FBG sensing points, the FBG flexible sensor was fabricated by grooved bonding. The fabrication process is shown in Figure 14.

First, two rows of grooves, each with a radius of 1 mm, were dug on the surface of the flexible rod at an interval of 180° along the axis direction (see Figure 14a). The two rows of grooves were sanded with fine-grit sandpaper until the surface was smooth, and then the grooves were cleaned with alcohol (see Figure 14b). The FBG sensing arrays were put into the two rows of grooves of the flexible rod and were fixed with POM glue. The distance between two adjacent sensing points in each groove was 50 mm. The two rows of grooves containing the sensing arrays were then sealed with waterproof glue, ensuring a smooth surface of the flexible rod after sealing (see Figure 14c). The use of POM glue to fix the FBG sensing array will not change the bending characteristics of the flexible substrate itself. Sealing the grooves with waterproof glue not only protects the sensing points, but also improves the strain-transfer rate between the sensing points and flexible substrate. Finally, the pigtail was drawn out from the end of the flexible rod. To protect the fragile fiber, a loose fiber tube with a diameter of 0.9 mm was used for protection (see Figure 14d). Because the FBG sensing arrays were arranged at 180° intervals, the difference between the central-wavelength changes of the two sensing points in the same detection unit can be used to eliminate the influence of temperature in the experiment. Therefore, the designed FBG flexible sensor has temperature self-compensation characteristics.

### 4.2. Deformation-Sensing Experiment and Analysis

**Figure 15:**
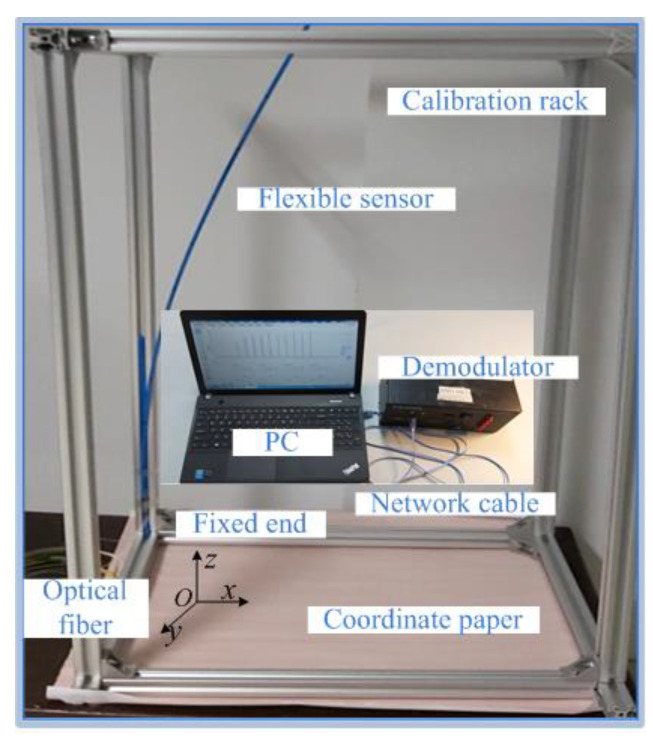
Diagram of deformation-sensing experimental device.

The FBG flexible sensor was designed to encapsulate the sensing points in grooves with a radius of 1 mm by means of grooved bonding. The strain information of the detection unit in the displacement-field reconstruction was obtained by using the difference in the wavelength variations before and after the deformation of the flexible sensor. These measures not only increase the strain-transfer rate between the flexible substrate and sensing points, but also reduce the influence of prestrain and dislocations on the reconstruction accuracy of the deformation field. Deformation-sensing experiments were carried out on the designed FBG flexible sensor to verify the feasibility and effectiveness of the simulation theory. The experimental system is shown in Figure 15. The bottom end of the sensor was fixed on the calibration rack, and the bottom of the calibration rack was covered with millimeter-squared coordinate paper. It was assumed that the height direction of the flexible sensor was the *z*-axis, the plane covered with the coordinate paper was the *x*–*O*–*y* plane, and the coordinate of the center point of the fixed end was *O* (0, 0, 0). The coordinates of the sensing points along the *z*-axis were *S_i_* (± 2.5, 0, 25 + 50*i*), where *i* = 0, 1, …, 39; the coordinates of the measuring points were *M_j_* (2.5, 0, 50*j*), where *j* = 1, 2, …, 40. One end of the flexible sensor led out a pigtail and was connected to the demodulator, and the wavelength data of the sensing points acquired by the demodulator were transmitted to the computer by the network cable. When the FBG flexible sensor was in a free state, the central wavelength of each sensing point was collected as the initial wavelength. Displacements of 150 mm, 50 mm, and 120 mm were applied along the *x*-axis direction at the (−2.5, 0, 600), (−2.5, 0, 1300), and (−2.5, 0, 2000) coordinates of the flexible sensor, respectively. After the flexible sensor was stabilized, the displacement sensor was used to read the displacement value of each measuring point along the *x*-axis direction. At this time, the demodulator collected the central wavelength of each sensing point as the current wavelength. The difference between the current wavelength and corresponding initial wavelength was the strain information of each detection unit. The reconstructed displacement of the flexible sensor can be obtained by using the displacement reconstruction algorithm based on the strain information. Three repeated experiments were performed for the deformation sensing of flexible sensors, and the applied data were the averages of the three experiments.

**Figure 16:**
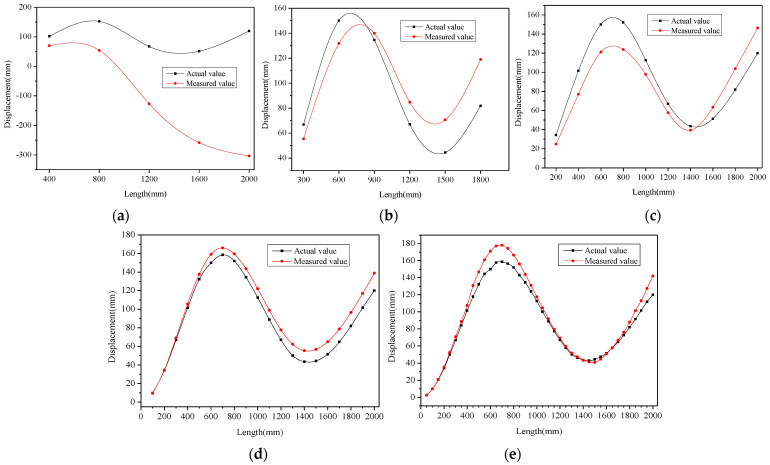
Comparison between calculated displacement and measured displacement of measuring points at different intervals: (**a**) *l* = 400 mm; (**b**) *l* = 300 mm; (**c**) *l* = 200 mm; (**d**) *l* = 100 mm; (**e**) *l* = 50 mm.

The strain value of the sensing point in the flexible sensor was extracted along the *z*-axis direction with a distance interval of *l*. Let *l* be 400 mm, 300 mm, 200 mm, 100 mm, and 50 mm, which represent the layout interval of the sensing points, and then the arc-fitting algorithm can be used to reconstruct the shape of the flexible sensor. As shown in Figure 16, the displacement of the measuring point reconstructed by the algorithm was compared with the measured displacement at different intervals. It can be seen that there were errors in both the reconstructed displacement and measured displacement of the measuring points, but with the decrease in the layout interval of the sensing points, the coincidence degree of the two curves in the figure will be higher. The average absolute errors of the reconstructed measurement points at different intervals were 211.32 mm (*l* = 400 mm), 19.36 mm (*l* = 300 mm), 17.96 mm (*l* = 200 mm), 9.40 mm and (*l* = 100 mm), and 7.12 mm (*l* = 50 mm). Therefore, the deformation-sensing accuracy of the flexible sensor can be improved by reducing the arrangement interval of the sensing points. However, the spacing of the sensor points cannot be infinitely small, and it was also limited by the following factors. The displacement reconstruction algorithm generally requires that the arrangement interval of the sensing points is equal. FBG strings were used in the fabrication of the FBG flexible sensors. After the FBG strings were written, the interval between the sensing points was constant. However, the grating region has a certain length, and the length of the grating region affects the reflectivity of the spectrum. Therefore, the algorithm was not applied in the paper to optimize the spacing *l* of the sensing points. In this experiment, the FBG string with a grating length of 10 mm was selected. Factors such as the fabrication cost of the sensor, the strain-transfer rate between the sensing points and flexible substrate, and the ease of the demodulation of the central wavelength of the sensing points need to be considered. In practical applications, the layout interval of sensing points is generally 100 mm. In this case, the average absolute error of the reconstructed measuring points in the experiment was 9.40 mm. Affected by experimental conditions and other factors, the error value obtained is slightly larger than the simulation result (7.12 mm), but this index value can meet the needs of engineering monitoring.

## 5. Conclusions

In this paper, theoretical derivation and model establishment were adopted, and the referential parameters are given. The effects of the length, width, and thickness of the cemented layer, the shear modulus of the flexible substrate, and the radius of the groove on the strain-transfer rate were analyzed. To improve the strain-transfer rate between the sensing point and flexible substrate, the length of the cemented layer should be increased as much as possible when the installation space allows. The width and thickness of the cemented layer, the shear modulus of the flexible substrate, and the groove radius are about 3 mm, 1 mm, 1 GPa, and 1 mm, respectively. The displacement reconstruction algorithm is essentially a recursive algorithm, which inevitably introduces accumulated error. The influence of the point spacing on the measurement accuracy of the flexible sensor was analyzed by finite element simulation. Considering the manufacturing cost and allowable error range of the sensor, a layout interval of 100 mm sensing points was chosen. To protect the fragile FBG sensing points, a flexible FBG sensor was fabricated by means of grooved bonding. The error law of the reconstructed measuring points in the deformation-sensing experiment was consistent with the simulation results. When the layout intervals of the sensing points were 100 mm, the average absolute error of the reconstructed measuring points in the experiment was 9.40 mm. Affected by experimental conditions and other factors, the error value obtained is slightly larger than the simulation result (7.12 mm), but this index value can meet the needs of engineering monitoring. Deformation-sensing experiments verified the feasibility and effectiveness of the simulation theory. The research results can theoretically guide the packaging and fabrication of the FBG flexible sensor, thereby improving the measurement accuracy of the sensor for the measured structure.

## Figures and Tables

**Table 1:** Parameter-value range of each physical quantity.

Physical Quantity	Parameter Value
2*L_f_*	>10 mm
*D_c_*	1–5 mm
*h_c_*	0.4–2 mm
*E_c_*	1–10 GPa
*G_c_*	0.4–2 GPa
*G_s_*	0.1–2 GPa
*h_d_*	0.5 mm

**Table 2:** Comparison of maximum-absolute-error value and maximum-error percentage under different intervals.

Extraction Interval	Maximum Absolute Error	Maximum-Error Percentage
400 mm	409.05 mm	582.92%
300 mm	36.12 mm	55.90%
200 mm	34.07 mm	35.96%
100 mm	13.56 mm	11.30%
50 mm	8.73 mm	8.84%

**Table 3:** Detailed parameters of fiber-grating sensing arrays.

Parameters	Description
Fiber type	Single-mode fiber
Elastic modulus	72 GPa
Grating length	10 mm
Bandwidth (3 dB)	<0.2 nm
Side-lobe-suppression ratio	>15 dB

## Data Availability

Not applicable.
